# Evaluation of Antioxidant and Anti-Glycemic Characteristics of Aged Lemon Peel Induced by Three Thermal Browning Models: Hot-Air Drying, High Temperature and Humidity, and Steam-Drying Cycle

**DOI:** 10.3390/foods13193053

**Published:** 2024-09-25

**Authors:** Kai-Chun Chuang, Yi-Chan Chiang, Yi-Jou Chang, Yen-Chieh Lee, Po-Yuan Chiang

**Affiliations:** Department of Food Science and Biotechnology, National Chung Hsing University, Taichung 40227, Taiwan

**Keywords:** aged lemon peel, moisture-assisted aging technology, antioxidant activity, α-glucosidase inhibition

## Abstract

This study evaluated the antioxidant and anti-glycemic properties of black lemon Chenpi (BLC) (*Citrus limon* (L.) Burm. f. cv. Eureka), processed using three thermal browning models—hot-air drying (HAL), high temperature and humidity, and steam-drying cycle (SCL)—and compared them to fresh lemon peel and commercial Chenpi. The moisture-assisted aging technology (MAAT) is an environmentally friendly process for inducing browning reactions in the lemon peel, enhancing its functional properties. Our results demonstrated significant increases in sucrose, total flavonoid content, and antioxidant capacities (2,2-diphenylpicrylhydrazyl: 12.86 Trolox/g dry weight; ferric reducing antioxidant power: 14.92 mg Trolox/g dry weight) with the MAAT-HAL model. The MAAT-SCL model significantly improved the browning degree, fructose, total polyphenol content, narirutin, and 5-hydroxymethylfurfural synthesis (*p* < 0.05). Additionally, aged lemon peel exhibited potential α-glucosidase inhibitory activity (28.28%), suggesting its role in blood sugar regulation after meals. The multivariate analysis (principal component and heatmap analyses) indicated that BLC processed using the MAAT-SCL model exhibited similarities to commercial Chenpi, indicating its potential for functional food development. Our results indicate that MAAT-SCL can enhance the economic value of lemon by-products, offering a sustainable and functional alternative to traditional Chenpi.

## 1. Introduction

Lemon is one of the most important fruits worldwide. It is rich in various functional components, such as flavonoids, vitamins, carotenoids, low-molecule phenolic acids, and essential oils [1]. It also has physiological functions, such as anti-inflammatory and anti-cancer activities and preventing cardiovascular diseases [2,3]. Its main uses in the food industry include juice production and essential oil extraction, of which 50–60% of by-products are derived, including peels, seeds, and epidermal membranes [4,5]. *Citrus limon* (L.) Burm. f. cv. Eureka is widely accepted in Taiwan for its unique aroma and flavor. It complies with the sustainable development goals (SDGs) through clean energy and by-product reuse [6,7].

Moisture-assisted aging technology (MAAT) is a green processing technology that gives Chenpi a unique flavor and deeper color through high temperature, high humidity, and prolonged thermal fermentation [8,9,10]. In addition, aging products, such as black garlic, black bitter melon, black apple, black tomato, and black orange, have improved antioxidant, anti-allergic, anti-diabetic, anti-inflammatory, blood-lipid-lowering, and anti-cancer activities. Non-enzymatic browning reactions during the aging process, such as the Maillard reaction and ascorbic acid degradation [7,8,9,10,11,12,13], synthesize 5-hydroxymethylfurfural (5-HMF) under an acidic environment. The intermediate product has been confirmed to have antioxidant and α-glucosidase inhibition activities, which improve blood sugar levels after meals [9,10,14]. However, ascorbic acid generates furfural during a specific aging process due to oxidation, raising a safety concern [15,16,17,18,19]. In addition, previous studies have highlighted that aging reactions can increase free-form phenolic acids to improve functionalities and free-form carotenoids to improve extraction efficiency and hydrolyze glycosidic bonds to increase monosaccharide concentration and sweetness [9]. Among the aging reactions under different thermal treatment models, the steaming-drying aging cycle model has been indicated to shorten the procedure by 33%, which is closest to the SDGs of sustainable development, improved health and well-being and energy efficiency [10].

In addition, using by-products for functional material development is the best choice for a sustainable economy [3,20,21]. Like citrus peel (Citri Reticulatae Pericarpium), lemon peel is rich in various polyphenols, flavonoids, terpenes, limonoids, lower molecular weight phenolic acids, and organic acids, such as eriocitrin, hesperidin, limonene, and malic acid [22,23,24]. A previous study found that the sensory properties of Eureka black lemon slices manufactured at high temperature and humidity (HHL) were similar to those of commercial Chenpi and highly marketable among consumers. The browning reaction of black lemon slices increases total polyphenols, flavonoids, and antioxidant activities while reducing limonin levels, thereby increasing palatability [7].

There is currently no relevant research on using lemon peel to produce commercial Chenpi-like functional aging products. Therefore, our goal was to enhance the value of lemon by-products through an artificial aging process that utilizes clean energy. This study conducted a primary assessment of the hesperidin content of Chenpi and further evaluated the effects of thermal treatments on the physicochemical properties, browning properties, and functionalities of black lemon Chenpi (BLC) in different aging models—HAL, HHL, and SCL—compared to fresh lemon peel. Finally, principal component and heatmap analyses were conducted to compare the commercial accessibility of BLC and commercial Chenpi.

## 2. Materials and Methods

### 2.1. Materials and Chemicals

Each group of fresh lemons (*Citrus limon* (L.) Burm. f. cv. Eureka) weighed 110 ± 15 g and were purchased from Pingtung Lemon Marketing Cooperative (Pingtung County, Taiwan). Lemon peels were stored at 4 °C after being transported, sorted, rinsed, and peeled at 25 ± 5 °C. All chemicals were supplied from Sigma-Aldrich Co. (St. Louis, MO, USA). Deionized water with a resistivity of not <18.2 MΩ cm^−^^1^ was used to prepare all solutions. The “Chenpi” merchandise samples were purchased from local Chinese medicine stores (Taichung City, Taiwan). Sample FJ, CT, and XC was purchased from Fuji Chinese medicine stores, Cintong Chinese medicine stores, and Xinchuang Chinese medicine stores, respectively.

### 2.2. Preparation of BLC

Fresh lemon peels (FL) were separated into four groups (Table 1). Hot-air drying (HAD) was performed with a dehydrator (Model 3926 TB, Excalibur Dehydrator Co., Sacramento, CA, USA) and a temperature and humidity chamber (Model BTH80/20, Firstek Co., New Taipei, Taiwan), and a steamer (RUEY MENG METAL CO., LTD, Tainan City, Taiwan) was used for the moisture-assisted aging technology (MAAT) [9]. The hot-air-dried BLC (HAL) group was processed by HAD directly at 55 °C for 15 days. The steam-drying cycle BLC (SCL) was steamed in a boiled steamer for 1 h and then immediately treated with HAD at 55 °C for 5 h. After that, the SCL was stored at room temperature (25 ± 5 °C) for 48 h. This cycle was repeated five times. The high-temperature and -humidity BLC (HHL) group underwent direct MAAT processing within fifteen days at 55 °C and 75% relative humidity (RH).

### 2.3. Quality Indices of BLC

#### 2.3.1. Appearance

Following the method of Lin et al. [25], a stereoscopic dissecting microscope (Model SMZ800, Nikon Co., Tokyo, Japan) and a digital single-lens reflex camera (Model 450D, Canon Co., Tokyo, Japan) were used to photograph the surface microstructure of BLC.

#### 2.3.2. Color Analysis

The lightness value (L*), red/green coordinate (a*), and yellow/blue coordinate (b*) of BLC were measured with a color meter (Model ZE-2000, Nippon Denshoku Industries Co., Tokyo, Japan) and calibrated with a standard whiteboard (X = 92.81, Y = 94.83, Z = 111.71) and a zero box. The calculation of the color difference (ΔE) followed the formula below (Equation (1)).

ΔE = [(L*_1_ − L*_0_)^2^ + (a*_1_ − a*_0_)^2^ + (b*_1_ − b*_0_)^2^]^0.5^ (1)

#### 2.3.3. Water Activity and Moisture Content

A water activity meter (Model Aqualab 3TE, Meter Group Inc., Pullman, WA, USA) assessed water activity of BLC. Following the moisture examination was A.O.A.C. 32.1.02 (2000). One g of sample (W) was weighed into a constant-weight weighing bottle (W_1_) and moved it into an oven (Model OV-23, TSAO HSIN ENTERPRISE CO., Taichung City, Taiwan) to dry at 105 °C to a constant weight (W_2_). The calculation formula is as follows: The moisture content was calculated by Equation (2).

Moisture content (%) = [W − (W_2_ − W_1_)]/W (2)

### 2.4. Physical Characteristics of BLC

A 1 g amount of dry sample powder was mixed with 20 mL of deionized water and ultrasonically extracted for 30 min using a vibrator. The liquid was filtered via a 0.22 µm PTFE filter (Waters Co., Milford, MA, USA). The BLC extracts were kept at −20 °C before analysis.

#### 2.4.1. Browning Degree

Following and modifying the method of Chiang and Chiang [9], BLC extracts were diluted with deionized water and measured at 420 nm with a microplate reader (Model SPECTROstar Nano, BMG Labtech Co., Ortenberg, Germany).

#### 2.4.2. pH Value

According to Huang et al. [26], BLC extract pH was measured with a pH meter (Model SP-2300, Suntex Instruments Co., New Taipei, Taiwan) after three-point calibration (pH 4, 7, and 10).

### 2.5. Chemical Composition of BLC

According to Chiang and Chiang [9], to extract 1 g of dried sample, mix with 25 mL of 70% LC-grade ethanol and utilize an ultrasonic vibrator for 1 h at 25 ± 5 °C. BLC extract supernatants were filtered using a 0.22 µm PTFE filter (Waters Co., Milford, MA, USA) to exclude precipitation before analysis.

#### 2.5.1. Sugar Composition Analysis

A chromatographic pump (Model Chromaster 5110, Hitachi Co., Tokyo, Japan), a Shodex RSpak DC-613 column (150 mm, 6 mm i.d.) (Kanto Co., Tokyo, Japan), and a refractive index detector (Model 5450) make up a high-performance liquid chromatography with refractive index detector (HPLC-RI) system. Analyzed for 20 min, the mobile phase used the isocratic of acetonitrile (80%) with 1.5 mM NaOH in distilled water (20%); 15 μL samples were injected at 70 °C with a 1.50 mL/min mobile-phase flow rate.

#### 2.5.2. Maillard Reaction Products Analysis

The high-performance liquid chromatography with diode array detector (HPLC-DAD) system included a chromatographic pump (Model Chro-master 5110, Hitachi Co., Tokyo, Japan), a Mightysil RP-18GP column (250 mm, 4.6 mm i.d., 5.0 μm) (Kanto Co., Tokyo, Japan), and a diode array detector (Model L-2450, Hitachi Co., Tokyo, Japan). A 20 min analysis was performed on the same 12% acetonitrile gradient in distilled water, and 15 μL samples were injected at 25 °C with a 1.00 mL/min mobile phase flow rate. The detective wavelength for 5-hydroxymethylfurfural and furfural was 284 nm (Figure S1).

#### 2.5.3. Polyphenol Analysis

Following and modifying Hsu et al. [10], we used the same HPLC-DAD system as Section 2.5.2. The mobile phase was 0.1% formic acid (A) and acetonitrile (B), and the dilution gradient was 0–5 min; 5–20 min: 63% A, 20–25 min: 50% A, 25–30 min: 20% A, 30–35 min: 0% A, 35–40 min: 63 A, 40–45 min: 95% A. The flow rate was 0.8 mL/min, and the injection volume was 10 µL. The detective wavelength for eriocitrin, narirutin, hesperidin, and nobiletin was 283 and 330 nm (Figure S1).

### 2.6. Fourier-Transform Infrared Spectroscopy of BLC

According to Yuan et al. [27], all BLC were examined using a Fourier-transform infrared spectrometer (FTIR) (Model Nicolet 6700, Thermo Fisher Scientific Co., Waltham, MA, USA) with an MCT detector. The scan ranged from 650 to 4000 cm^−^^1^. Spectra were collected at 2 cm^−1^/30 s resolution.

### 2.7. Antioxidant Contents and Activities Analysis of BLC

#### 2.7.1. Total Polyphenol and Flavonoid Contents

We followed the method by Hsu et al. [10]. For total polyphenol content (TPC) analysis, 70 μL BLC extracts were mixed with equal volumes of Folin–Ciocalteu reagent, vortexed, and stored in darkness for 3 min. Additionally, 35 μL of 10% Na_2_CO_3_ was vortexed and kept in distilled water for 30 min. A microplate reader (Model SPECTROstar Nano, BMG Labtech Co., Ortenberg, Germany) measured absorbance at 735 nm. Calibration curves measured gallic acid to dried weight (mg/g D.W.) (Figure S1).

For total flavonoid content (TFC) measurement, 10 μL of the BLC extracts were mixed with 60 μL distilled water, 30 μL 5% NaNO_2_ in double-distilled water, and stored in darkness for 6 min. We stored 25 μL of 25% AlCl_3_ in distilled water, 25 μL of 2% NaOH in double-distilled water, and 50 μL of distilled water for 15 min after vortexing. A microplate reader measured absorbance at 415 nm. Standard quercetin was quantified to dry weight (mg/g D.W.) by calibration curves (Figure S1).

#### 2.7.2. 2,2-Diphenyl-1-Picrylhydrazyl (DPPH) Radical-Scavenging Activity and Ferric Ion-Reducing Antioxidant Power (FRAP) of BLC

We followed and modified the method of Huang et al. [28]. For DPPH analysis, 10 μL of the BLC extracts were mixed with 40 μL of 100 mM Tris-HCl buffer (pH 7.4) and 75 μL 0.5 mM DPPH in LC-grade methanol and stored in darkness for 30 min. The absorbance value (A) was measured at 517 nm using a microplate reader.

The FRAP reagent was mixed with 300 mM acetate buffer (pH 3.6), 10 mM TPTZ in 40 mM HCl, and 20 mM FeCl_3_ in distilled water at a proportion of 10:1:1. Then, 20 μL of the BLC extracts were mixed with 150 μL of FRAP reagent and kept at 37 °C for 10 min in the dark. A microplate reader measured absorbance at 593 nm. Standard Trolox was measured in mg/g dried weight (mg/g D.W.) (Figure S1).

### 2.8. In Vitro Inhibitory Capacity of α-Glucosidase

According to Hsu et al. [10], 50 μL of the BLC extracts were combined with 100 μL α-glucosidase diluent (1.000 U/mL) and 50 μL p-NPG. At 25 ± 5 °C, the mixture was left in the dark for 5 min. The absorbance was determined at 400 nm using a microplate reader. The positive control for BLC extract inhibitory capability was 7 mg of acarbose diluted with 100 mL of 70% ethanol. The background group contained 50 μL of BLC extracts, 100 μL of PBS solution, and 50 μL of p-NPG. The PBS solution was composited with 7.7 g NaCl, 0.7 g Na_2_HPO_4_, and 0.2 g KH_2_PO_4_ in double-distilled water and pH 7.4-adjusted with 1 N NaOH. The BLC inhibitory rate was calculated using Equation (3).

α-Glucosidase inhibitory capacity (%) = (A_sample_ − A_background_)/(A_acarbose_ − A_background_) (3)

### 2.9. Statistical Analysis

All database results (n = 3) are shown as mean ± standard deviation. Data were gathered using SPSS version 12.0’s one-way ANOVA (IBM Co., Armonk, NY, USA). Statistical significance was established using Duncan’s multiple range test (DMRT), with *p* < 0.05 for all comparisons. XLSTAT software (XLSTAT 2023.3.0 (1415), Addinsoft Co., Long Island City, NY, USA) was used to construct the principal component analysis (PCA), agglomerative hierarchical clustering (AHC) and heatmap analysis (HA) according to Chiang and Chiang [9] and Chan et al. [29]’s statistical model.

## 3. Results and Discussion

### 3.1. Appearance and Physical Characteristics of BLC and Commercial Chenpi

**Figure 1:**
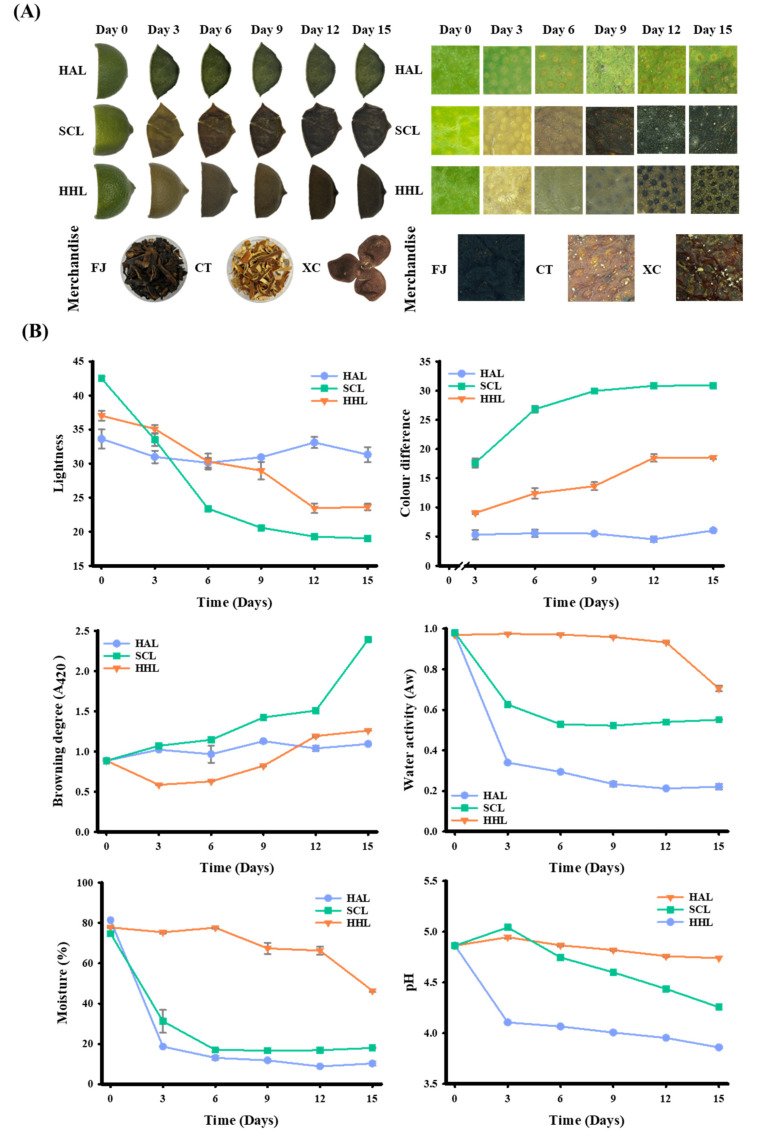
Effects of physical properties on black lemon Chenpi under different aging models. (**A**) Appearance and microstructure, (**B**) physical properties. L, F, HA, SC, and HH represent the lemon peel samples, fresh, hot-air drying, steam-drying cycle, and high temperature and humidity, respectively. FJ, CT, and XC were commercial Chenpi samples.

After three days of aging, the HAL- and SCL-treated BLC began to shrink due to water loss [30]. In addition, due to the softening of the peel tissue during the steaming process, the cell wall of the SCL-treated BLC was destructured. The shrinkage became more evident with aging time [31]. In comparison, the HHL model aged the peel in a high temperature (55 °C) and humidity environment (75% RH), and the moisture in the peel was maintained at a higher level (46.39–77.80%). The moisture was not easily removed, and the peel’s appearance differed from that of unprocessed peel [10]. The microstructure of the lemon peel revealed a greater presence of oil cells (Figure 1A), which was noted by Sun et al. [7]. After 15 days of MAAT, the L* ranged from 33.63 to 31.32 for HAL-treated BLC, 42.50 to 19.00 for SCL-treated BLC, and 37.03 to 23.63 for HHL-treated BLC (*p* < 0.05; Figure 1B). The ΔE was 6.04, 30.87, and 18.55 for the HAL-, SCL-, and HHL-treated BLC, respectively. Aging was significantly greater when MAAT was combined with SCL and HHL, indicating that these conditions accelerate the browning reaction rate [8,9,10]. Browning degree analysis (A_420_) can be used to evaluate the intermediates of the Maillard reaction. The HAL-, SCL-, and HHL-treated BLC showed upward trends of 1.094 ± 0.041, 2.395 ± 0.029, and 1.260 ± 0.002, respectively (*p* < 0.05). Hsu et al. [10] and Wang et al. [32] reported upward trends in similar aging treatments.

MAAT induces a browning reaction, and Aw is a crucial index that influences the rate of this reaction [10,33]. The Aw of BLC showed a similar trend to the water content. After MAAT for 15 days, the Aw and water contents of HAL-, SCL-, and HHL-treated BLC were significantly reduced compared to FL peel (Aw: from 0.974 to 0.221, 0.969 to 0.551, and 0.980 to 0.705, respectively; water content: from 81.37% to 10.25%, 77.78% to 17.99%, and 74.75% to 46.39%, respectively). Because peels are treated with HAD in the HAL and SCL models, their water loss is accelerated [30]. However, the Aw and water contents of SCL- and HHL-treated BLC are higher than those of HAL-treated BLC due to the ability of MAAT to balance the peel moisture, which in turn increases the browning reaction rate [9,10]. The pH of HAL-, SCL-, and HHL-treated BLC showed a downward trend with increasing MAAT time. During the aging process, it may be related to the carboxyl groups exposed by amino sugar conjugation during the Maillard reaction and the increase in lower-molecular-weight phenolic acids [13,34].

Three types of commercial Chenpi (FJ, CT, and XC) were collected. The L* was approximately 18.65–29.45, a* was 1.70–8.48, b* was 1.93–8.95, and A_420_ was 1.124–2.980. The Aw and water contents were approximately 0.532–0.608 and 14.58%–17.29%, respectively. Previous studies indicated that storage stability occurs when the water activity is <0.61 or the water content is <20% (Table 2) [33,35,36].

### 3.2. FTIR Spectrum of BLC and Commercial Chenpi

**Figure 2:**
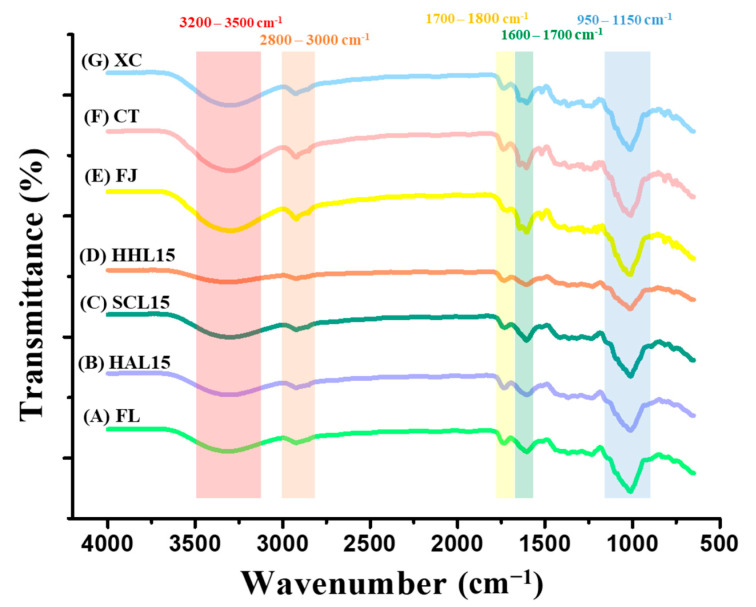
FTIR spectrum of black lemon Chenpi under different aging models and commercial Chenpi. L15, F, HA, SC, and HH represent the lemon peel samples with fifteen days’ process of fresh, hot-air drying, steam-drying cycle, and high temperature and humidity, respectively. FJ, CT, and XC were commercial Chenpi samples.

FTIR was used to measure the strength of functional groups, identify changes in characteristic bonding during processing, and speculate on the aging reaction mechanism [10]. The FTIR spectra of FL, BLC, and commercial Chenpi are shown in Figure 2. The peak at 3200–3500 cm^−1^ reflects hydroxyl group (−OH) stretching, representing the phenolic compounds and other hydroxyl-containing compounds [37]. HHL-treated BLC aged for 15 days (HHL15) has a lower peak value, which was attributed to the lower content of polyphenols and 5-HMF (Table 3 and Table 4). The peaks at 1700–1800 cm^−1^ and 1600–1700 cm^−1^ are the characteristic peaks of carbonyl group (−C=O) and aromatic group (C=C) stretching, respectively. The weaker bonding strength of HHL15 may be due to long-term MAAT accelerating the oxidation reaction and degrading alkenes, aldehydes, ketones, and esters [10,38]. The stretching of the ether bond (C−O−C), used to represent the glycosidic bonds in BLC (950–1150 cm^−1^), was weaker for HAL15 and HHL15, which was attributed to the MAAT increasing the kinetic energy of water and accelerating the glycosidic bond cleavage, thereby releasing more monosaccharides and increasing sweetness [10,39]. In addition, the peak at 1000–1100 cm^−1^ represented the stretching of the carbon–oxygen bond (–CO) and overlapped the wavelength of glycosidic bond stretching, which was enhanced in SCL15 due to the 5-HMF synthesized by the Maillard reaction during MAAT (Table 3) [40].

Commercial Chenpi (FJ, CT, and XC) had specific characteristic peaks at 3200–3500 and 2800–3000 cm^−1^ compared to FL and BLC, representing the hydroxyl group (−OH) and hydrocarbyl group (−CH), respectively. The stretching indicated greater contents of phenolic compounds and polyhydrocarbyl-substituted compounds (e.g., flavanone glycosides) (Table 4) [37,40]. Compared to BLC, commercial Chenpi had three characteristic peaks at 1600–1800 cm^−1^, representing the aromatic groups in polycarbonyl compounds (e.g., flavonoids) and the vibration of the carbonyl group in hemicellulose [38,41].

### 3.3. Chemical Composition of BLC and Commercial Chenpi

Compared to FL peel, the sucrose content of HAL-treated BLC was significantly higher because HAD treatment concentrated the matrix and reduced the rate of glycosidic bond cleavage [9,42,43]. The sucrose content of SCL15 and HHL15 decreased from 40.67 to 0.05 and 0.06 mg/g D.W., respectively (*p* < 0.05). The fructose content showed a slight upward trend in the middle and final stages of MAAT (BLC9, BLC12, and BLC15), which was attributed to glycosidic bond cleavage converting disaccharide into glucose and fructose and subsequent glucose isomerization into fructose [9,10,44]. The sucrose, glucose, and fructose contents of commercial Chenpi were 0.05–58.93, 57.94–64.39, and 61.46–90.05 mg/g D.W., respectively (Table 3).

The synthesis of 5-HMF began to be detected in SCL- and HHL-treated BLC after 9 and 15 days of MAAT, respectively, and its content increased from 0.22 to 1.96 and 0.09 mg/g D.W., respectively (*p* < 0.05; Table 3), which was related to water modulation. Under MAAT and HAD treatment, the Aw was approximately 0.551–0.974 (Table 2), accelerating non-enzymatic browning and the Maillard reaction [9,45]. These results appealed to the color and carbohydrate changes mentioned above (Figure 1 and Table 3). Furfural has been classified as possibly carcinogenic to humans (Group 2B) by the International Agency for Research on Cancer [18]. Our study did not detect furfural in BLC but did in FJ, the commercial Chenpi (0.04 mg/g D.W.). These results illustrate the potential safety of BLC.

### 3.4. Polyphenols of BLC and Commercial Chenpi

Citrus flavonoids are mainly composed of polymethoxy flavones and flavanone glycosides. Eriocitrin and hesperidin, two common functional compounds in lemons, have anti-mutagenic and cardiovascular prevention activities. Previous studies have reported the impact of free radical scavenging on diseases [42,46,47,48,49]. According to the Chinese Pharmacopoeia, hesperidin is a control indicator for the quality of Chenpi [47]. In recent years, studies have increasingly focused on the nobiletin content of Chenpi because it has antioxidant, anti-inflammatory, anti-cancer, and physiological activities [50,51,52].

The contents of eriocitrin, narirutin, and hesperidin in FL were 12.12, 1.73, and 49.36 mg/g D.W., respectively (Table 4). During the HAL aging process, the contents of the three flavanone glycosides were 9.69–12.57, 1.55–2.03, and 48.65–58.64 mg/g D.W., respectively. Compared to the significant decrease in eriocitrin content, the hesperidin content increased significantly in HAL15 compared to FL peel due to the drying process at <100 °C retaining a higher content of flavanone glycosides [46]. The contents of eriocitrin and hesperidin in SCL15 and HHL15 decreased simultaneously since MAAT can cause the cell wall to destructure to release more polyphenols and further decompose into lower molecular weight phenolic acids [10,13]. The narirutin content of SCL15 and HHL15 was significantly and slightly increased, respectively (*p* > 0.05).

The nobiletin content of commercial Chenpi was approximately 0.22–4.51 mg/g D.W., which has antioxidant physiological functions. However, it was not detected in BLC, which can be attributed to species differences. In contrast, only trace amounts of eriocitrin were detected in commercial Chenpi (0.10–0.98 mg/g D.W.; *p* < 0.05), indicating that eriocitrin in BLC, which was developed from lemon peel, can be used as a unique functional material due to its antioxidant activities, neuroprotective effect, and ability to prevent metabolic syndrome [53,54,55].

### 3.5. Functionalities of BLC and Commercial Chenpi

**Figure 3:**
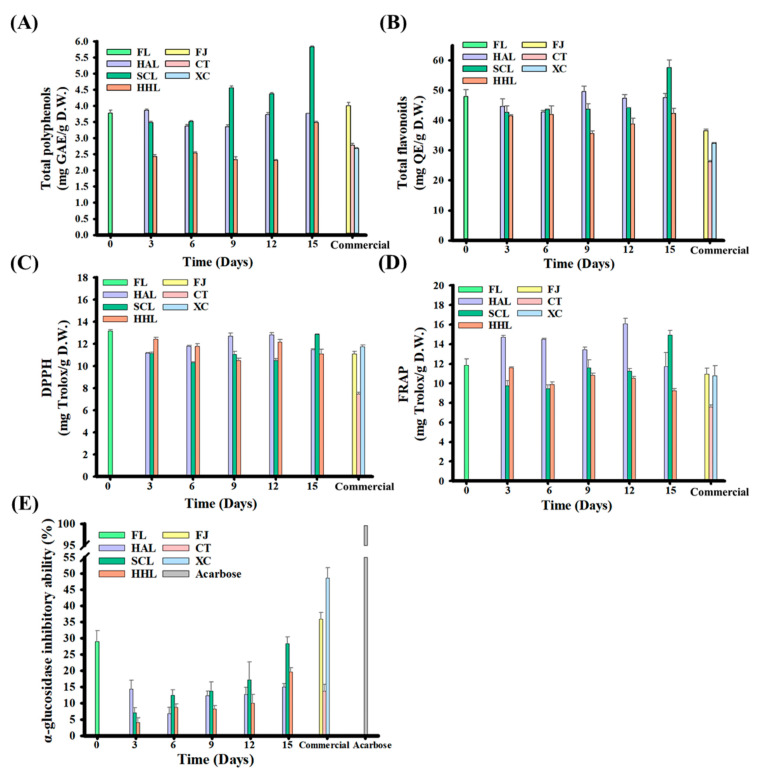
Functional characteristics of black lemon Chenpi under different aging models and commercial Chenpi. (**A**) Total polyphenols, (**B**) total flavonoids, (**C**) DPPH, (**D**) FRAP, (**E**) inhibition of in vitro α-glucosidase activity. L, F, HA, SC, and HH represent the lemon peel samples, fresh, hot-air drying, steam-drying cycle, and high temperature and humidity, respectively. FJ, CT, and XC were commercial Chenpi samples.

FL peel contains various physiologically active components, such as flavanone glycosides and lower molecular weight phenolic acids, which benefit human physiological health [21,24,43]. Previous studies have highlighted that citrus fruits have α-glucosidase inhibitory activity, which is mainly found in the villi of the small intestine and can decompose macromolecular sugars such as polysaccharides and disaccharides into glucose, thereby increasing postprandial blood glucose concentration [10,13,56]. The TPC and TFC contents were 3.77 mg GAE/g D.W. and 47.99 mg QE/g D.W. in FL peel, respectively, and 2.42–5.82 mg GAE/g D.W. and 41.49–57.52 mg QE/g D.W. in BLC, respectively (Figure 3A,B). Among them, TPC or TFC increased significantly with aging in SCL-treated BLC. The former was attributed to the MAAT with HHL that destructured BLC and improved the extraction efficiency of polyphenols; the latter explains the decomposition of flavonoid glycosides, such as rutin and hesperidin, which are hydrolyzed into quercetin and hesperetin, respectively, thereby increasing flavonoids [10,13,57].

The antioxidant capacities of DPPH and FRAP are widely used to evaluate the human diet. Antioxidants neutralize free radicals through electron conversion, prevent increased oxidative stress, improve the symptoms of chronic diseases, and reduce cell damage [10,58]. The DPPH and FRAP were 13.17 and 11.88 mg Trolox/g D.W. for FL peel, respectively, and 11.11–12.86 and 9.24–14.92 mg Trolox/g D.W. for BLC, respectively, after aging for 15 days (Figure 3C,D). The increases in total polyphenols and flavonoids did not increase the antioxidant activities, possibly due to the simultaneous consumption of antioxidants during the aging process and the reaction rate being higher than the antioxidant release rate during cell deconstruction. However, 5-HMF synthesized through aging increases the antioxidant capacities of SCL9, SCL12, and SCL15 (Table 3) [10,19,32].

Both FL and BLC15 could inhibit α-glucosidase activity, and their inhibition rates were approximately 28.88 and 14.97–28.28%, respectively (Figure 3E). This phenomenon may be because the polyphenols in lemon peel can bind to the active sites of α-glucosidase to block the combination of disaccharides, and polysaccharides compete for binding sites, thereby increasing the inhibition rate [59]. In addition, the α-glucosidase inhibitory activities of SCL- and HHL-treated BLC began to increase after three days of aging, increasing from 7.06% to 28.28% and 4.13% to 19.60% after 15 days of aging, respectively (*p* < 0.05). Previous studies indicated a positive correlation with the increase in 5-HMF concentration [9,10,13]. The α-glucosidase inhibition rate of commercial Chenpi was approximately 13.67 to 48.52% because it not only benefits from 5-HMF but also nobiletin [59].

### 3.6. Difference between Three Thermal Browning Models

**Figure 4:**
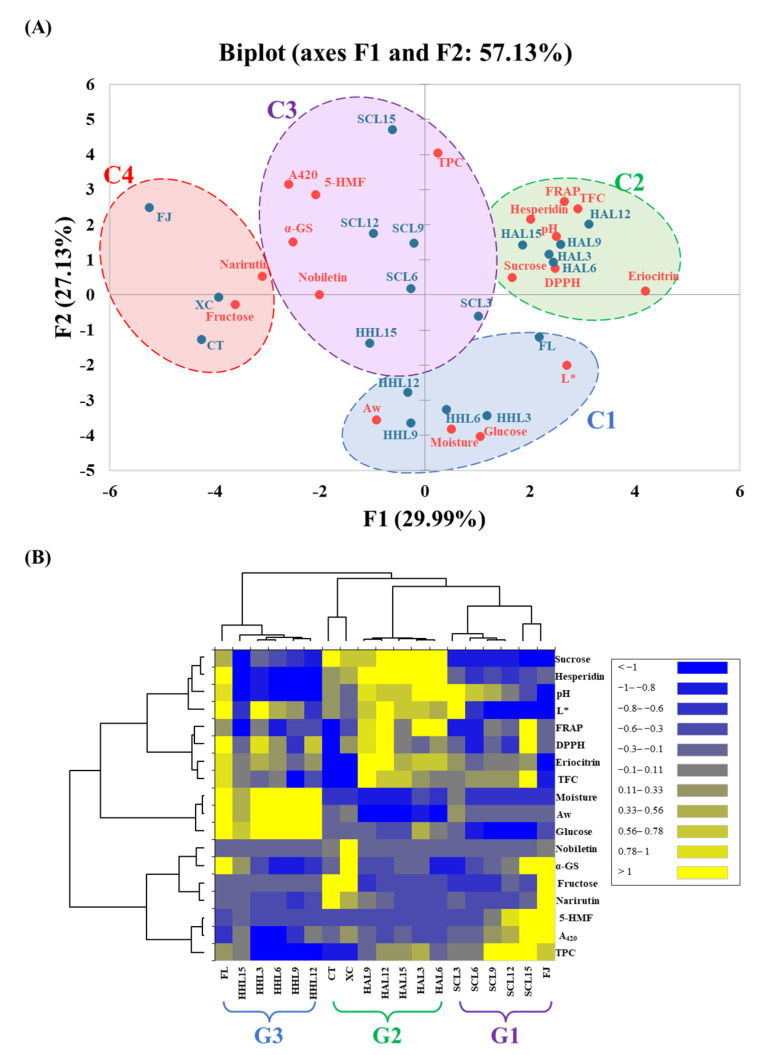
Multivariate analysis of black lemon Chenpi under different aging models and commercial Chenpi. (**A**) Principal component analysis. (**B**) Heatmap analysis. L, F, HA, SC, and HH represent the lemon peel samples, fresh, hot-air drying, steam-drying cycle, and high temperature and humidity, respectively. FJ, CT, and XC were commercial Chenpi samples.

In order to explore the physical and chemical properties and functional changes of BLC prepared using different aging models, PCA and heatmap analysis were used to clarify the relationship between the components in BLC. The PCA plot shows that the principal components F1 and F2 have 29.99 and 27.13% data representativeness, respectively (i.e., a total of 57.13%; Figure 4A). The AHC analysis divided all BLC samples into four major clusters that differed significantly (*p* < 0.05): C1 (FL, HHL3, HHL6, HHL9, and HHL12), C2 (HAL3, HAL6, HAL9, HAL12, and HAL15), C3 (SCL3, SCL6, SCL9, SCL12, SCL15, and HHL15), and C4 (FJ, CT, and XC).

C3 (Cluster 3) was located in the center of the PCA plots. It reflected a higher browning degree, higher 5-HMF and polyphenol content, and better α-glucosidase inhibitory activity, attributed to phenols, which decomposed into free-form polyphenols and synthesized 5-HMF during the MAAT process to improve the α-glucosidase inhibitory activity [10,13]. Cluster C2 was located on the right side of the PCA plots. It reflected higher sucrose, flavanone glycoside (eriocitrin and hesperidin), and TFC contents, and better DPPH and FRAP antioxidant activities. It was attributed to HAL reducing the moisture of the lemon peel and slowing the hydrolysis and oxidation reaction rates, keeping sucrose and flavanone glycosides at a higher level [9,42,43]. Flavanone glycosides have excellent antioxidant capacities, increasing the overall antioxidant capacity [47,48].

Cluster C1 was located at the bottom of the PCA plots. It reflected higher Aw, water content, and glucose content, attributed to water being an important substrate for chemical reactions, accelerating hydrolysis reactions under high-temperature conditions [9]. Cluster C4 was located on the left side of the PCA plots. It reflected higher fructose and narirutin contents, highlighting the various differences between commercial Chenpi and BLC.

In the heatmap, from right to left, were groups G1 (SCL3, SCL6, SCL9, SCL12, SCL15, and FJ), G2 (HAL3, HAL6, HAL9, HAL12, HAL15, CT, and XC), and G3 (FL, HHL3, HHL6, HHL9, HHL12, and HHL15). The heatmap enables the three different thermal browning models (HAL, SCL, and HHL) for preparing BLC to be quickly explored (Figure 4B). BLCs in G2 prepared through HAL showed higher flavanone content, sucrose content, and antioxidant activities, which were similar to those of commercial Chenpi (CT and XC). Due to the increases in lower molecular weight phenolic acid, fructose, and 5-HMF contents during the MAAT process, BLCs in G1 had higher polyphenol contents and inhibitory α-glucosidase activities, showing better functional characteristics. The high moisture content under HHL conditions promoted the glycosidic bond cleavage of sucrose in BLCs in G3, increasing their sweetness.

In summary, as a green process, combining MAAT with different aging models to prepare BLC can provide similar quality characteristics to commercial Chenpi and effectively shorten the manufacture time.

## 4. Conclusions

Our study demonstrated that employing MAAT with different thermal browning models greatly improved the antioxidant and anti-glycemic properties of lemon peel. We conducted rapid multivariate analysis to assess three models, finding that SCL produced the best results, yielding increased total polyphenols, flavonoids, and antioxidant activities and higher α-glucosidase inhibition. The synthesis of 5-HMF and undetected furfural further illustrates the safety and functional potential of BLC. Our results indicate the potential for developing aged lemon peel as a functional material for food industry applications, providing a valuable and sustainable use for lemon by-products that aligns with environmental sustainability and waste reduction goals.

## Figures and Tables

**Table 1:** Processing parameters of black lemon Chenpi under different aging models.

Group	HAD		MAAT	
Instruments	Dehydrator		Steamer	Humidity Chamber
Condition	55 °C, 15 Days	55 °C, 5 h	100 ± 2 °C, 1 h	55 °C, 75% RH, 15 Days
FL	-	-	-	-
HAL	⬤	-	-	-
SCL	-	⬤	⬤	-
HHL	-	-	-	⬤

HAD, MAAT, and RH represent the hot-air drying, moisture-assisted aging process and relative humidity, respectively. L, F, HA, SC, and HH represent the lemon peel samples, fresh, hot-air drying, steam-drying cycle, and high temperature and humidity, respectively. ⬤ and - represent the sample group that was processed with and without that treatment.

**Table 2:** Physical properties of black lemon Chenpi under different aging models and commercial Chenpi.

Day	Group	L*	a*	b*	A_420_	Water Activity	Moisture (%)	pH
0	FL	35.28 ± 1.70 ^f^	−6.82 ± 0.23 ^a^	14.37 ± 0.84 ^g^	0.884 ± 0.004 ^a^	0.974 ± 0.006 ^f^	78.31 ± 2.84 ^e^	4.86 ± 0.01 ^g^
15	HAL	31.32 ± 1.11 ^e^	−1.54 ± 0.15 ^b^	12.28 ± 0.29 ^f^	1.094 ± 0.041 ^b^	0.221 ± 0.014 ^a^	10.25 ± 0.74 ^a^	4.74 ± 0.00 ^f^
	SCL	19.40 ± 0.23 ^a^	2.38 ± 0.51 ^d^	3.11 ± 0.09 ^b^	2.395 ± 0.029 ^e^	0.551 ± 0.002 ^c^	17.99 ± 0.19 ^c^	4.26 ± 0.01 ^c^
	HHL	23.63 ± 0.46 ^b^	4.25 ± 0.49 ^e^	7.49 ± 0.33 ^d^	1.260 ± 0.002 ^c^	0.705 ± 0.013 ^e^	46.39 ± 0.44 ^d^	3.86 ± 0.00 ^a^
Commercial	FJ	18.65 ± 0.23 ^a^	1.70 ± 0.12 ^c^	1.93 ± 0.14 ^a^	2.980 ± 0.011 ^f^	0.536 ± 0.006 ^bc^	16.18 ± 0.43 ^bc^	3.89 ± 0.02 ^b^
	CT	26.84 ± 0.24 ^c^	8.48 ± 0.14 ^f^	8.95 ± 0.10 ^e^	1.124 ± 0.007 ^b^	0.532 ± 0.004 ^b^	14.58 ± 0.28 ^b^	4.51 ± 0.02 ^e^
	XC	29.45 ± 1.12 ^d^	2.74 ± 0.31 ^d^	3.81 ± 0.29 ^c^	1.362 ± 0.002 ^d^	0.608 ± 0.009 ^d^	17.29 ± 0.39 ^c^	4.40 ± 0.01 ^d^

Each value is expressed as mean ± standard deviation (n = 3); “^a–g^” means with different letters in the same column have significant difference (*p* < 0.05). L, F, HA, SC, and HH represent the lemon peel samples, fresh, hot-air drying, steam-drying cycle, and high temperature and humidity, respectively. FJ, CT, and XC were commercial Chenpi samples.

**Table 3:** Chemical composition of black lemon Chenpi under different aging models and commercial Chenpi.

Group	Day	Sucrose	Glucose	Fructose	5-HMF	Furfural
		(mg/g D.W.)				
FL	0	40.67 ± 1.69 ^f^	104.00 ± 1.22 ^l^	15.30 ± 0.42 ^fg^	ND	ND
HAL	3	66.75 ± 0.78 ^i^	79.76 ± 1.37 ^i^	10.72 ± 1.52 ^e^	ND	ND
	6	64.92 ± 0.34 ^i^	73.00 ± 1.69 ^h^	7.53 ± 1.42 ^cd^	ND	ND
	9	45.52 ± 1.60 ^g^	66.57 ± 2.13 ^g^	4.90 ± 0.75 ^abc^	ND	ND
	12	66.75 ± 7.43 ^i^	61.16 ± 0.86 ^f^	8.70 ± 0.51 ^de^	ND	ND
	15	59.52 ± 1.48 ^h^	59.1 ± 1.36 ^ef^	6.67 ± 0.48 ^bcd^	ND	ND
SCL	3	5.50 ± 0.23 ^b^	66.07 ± 0.54 ^g^	4.76 ± 1.57 ^ab^	ND	ND
	6	2.79 ± 0.70 ^ab^	50.87 ± 2.75 ^d^	3.38 ± 0.68 ^a^	ND	ND
	9	2.15 ± 0.43 ^ab^	43.81 ± 0.10 ^c^	4.93 ± 0.27 ^abc^	0.22 ± 0.00 ^b^	ND
	12	1.24 ± 0.79 ^a^	34.54 ± 0.95 ^b^	13.84 ± 1.37 ^f^	0.89 ± 0.00 ^c^	ND
	15	0.05 ± 0.39 ^a^	27.11 ± 2.33 ^a^	10.50 ± 2.41 ^e^	1.96 ± 0.01 ^d^	ND
HHL	3	22.04 ± 2.25 ^e^	113.46 ± 0.25 ^m^	13.37 ± 0.35 ^f^	ND	ND
	6	16.79 ± 1.99 ^d^	100.87 ± 1.42 ^k^	14.95 ± 2.46 ^fg^	ND	ND
	9	11.90 ± 2.30 ^c^	105.74 ± 0.02 ^l^	14.37 ± 0.91 ^f^	ND	ND
	12	3.85 ± 0.79 ^ab^	99.07 ± 1.17 ^k^	15.10 ± 1.05 ^fg^	ND	ND
	15	0.06 ± 0.03 ^a^	86.27 ± 2.26 ^j^	17.15 ± 0.24 ^g^	0.09 ± 0.02 ^a^	ND
FJ	-	0.05 ± 1.62 ^a^	57.94 ± 0.15 ^e^	61.46 ± 0.23 ^h^	2.03 ± 0.04 ^e^	0.04 ± 0.03 ^a^
CT		58.93 ± 0.65 ^h^	64.21 ± 0.04 ^g^	89.64 ± 2.57 ^i^	ND	ND
XC		47.55 ± 2.20 ^g^	64.39 ± 0.99 ^g^	90.05 ± 3.40 ^i^	ND	ND

Each value is expressed as mean ± standard deviation (n = 3); “^a–m^” means with different letters in the same column have significant difference (*p* < 0.05). “ND” means not detected. L, F, HA, SC, and HH represent the lemon peel samples, fresh, hot-air drying, steam-drying cycle, and high temperature and humidity, respectively. FJ, CT, and XC were commercial Chenpi samples.

**Table 4:** Polyphenol compounds of black lemon Chenpi under different aging models and commercial Chenpi.

Group	Day	Eriocitrin	Narirutin	Hesperidin	Nobiletin
		(mg/g D.W.)			
FL	0	12.12 ± 0.01 ^m^	1.73 ± 0.06 ^fg^	49.36 ± 0.32 ^i^	ND
HAL	3	10.81 ± 0.15 ^k^	1.66 ± 0.03 ^e^	53.18 ± 0.73 ^k^	ND
	6	10.99 ± 0.15 ^l^	1.64 ± 0.03 ^de^	48.65 ± 0.67 ^i^	ND
	9	12.57 ± 0.18 ^n^	2.03 ± 0.03 ^j^	51.76 ± 0.71 ^j^	ND
	12	12.06 ± 0.17 ^m^	1.81 ± 0.03 ^h^	58.64 ± 0.81 ^l^	ND
	15	9.69 ± 0.07 ^i^	1.55 ± 0.04 ^bc^	53.34 ± 0.31 ^k^	ND
SCL	3	9.19 ± 0.12 ^h^	1.73 ± 0.02 ^fg^	31.08 ± 0.39 ^f^	ND
	6	7.74 ± 0.10 ^d^	1.68 ± 0.02 ^ef^	24.62 ± 0.31 ^d^	ND
	9	8.38 ± 0.11 ^e^	1.65 ± 0.02 ^de^	28.84 ± 0.36 ^e^	ND
	12	8.74 ± 0.11 ^f^	1.51 ± 0.02 ^b^	25.47 ± 0.32 ^d^	ND
	15	9.15 ± 0.10 ^gh^	1.91 ± 0.09 ^i^	28.82 ± 0.38 ^e^	ND
HHL	3	10.27 ± 0.05 ^j^	1.60 ± 0.01 ^cd^	22.97 ± 0.10 ^c^	ND
	6	9.02 ± 0.04 ^g^	1.52 ± 0.01 ^b^	19.22 ± 0.09 ^b^	ND
	9	7.57 ± 0.03 ^c^	1.38 ± 0.01 ^a^	19.62 ± 0.09 ^b^	ND
	12	9.27 ± 0.04 ^h^	1.50 ± 0.01 ^b^	18.75 ± 0.08 ^ab^	ND
	15	8.72 ± 0.09 ^f^	1.75 ± 0.02 ^g^	18.22 ± 1.45 ^a^	ND
FJ	-	0.98 ± 0.03 ^b^	4.09 ± 0.03 ^l^	30.94 ± 0.09 ^f^	0.27 ± 0.10 ^a^
CT		0.10 ± 0.00 ^a^	5.37 ± 0.03 ^m^	37.72 ± 0.31 ^g^	0.22 ± 0.00 ^a^
XC		0.20 ± 0.01 ^a^	2.47 ± 0.05 ^k^	40.45 ± 0.51 ^h^	4.51 ± 0.12 ^b^

Each value is expressed as mean ± standard deviation (n = 3); “^a–n^” means with different letters in the same column have significant difference (*p* < 0.05). “ND” means not detected. L, F, HA, SC, and HH represent the lemon peel samples, fresh, hot-air drying, steam-drying cycle, and high temperature and humidity, respectively. FJ, CT, and XC were commercial Chenpi samples.

## Data Availability

The original contributions presented in the study are included in the article/Supplementary Material. Further inquiries can be directed to the corresponding author.

## Supplementary Materials

The following supporting information can be downloaded at: https://www.mdpi.com/article/10.3390/foods13193053/s1, Figure S1: Calibration curve of standards.
