# Supplementary figures and images for: Evaluation of Antioxidant and Anti-Glycemic Characteristics of Aged Lemon Peel Induced by Three Thermal Browning Models: Hot-Air Drying, High Temperature and Humidity, and Steam-Drying Cycle
Source: Foods. 2024 Sep 25;13(19):3053. doi: 10.3390/foods13193053 (PMC11475740; doi:10.3390/foods13193053)

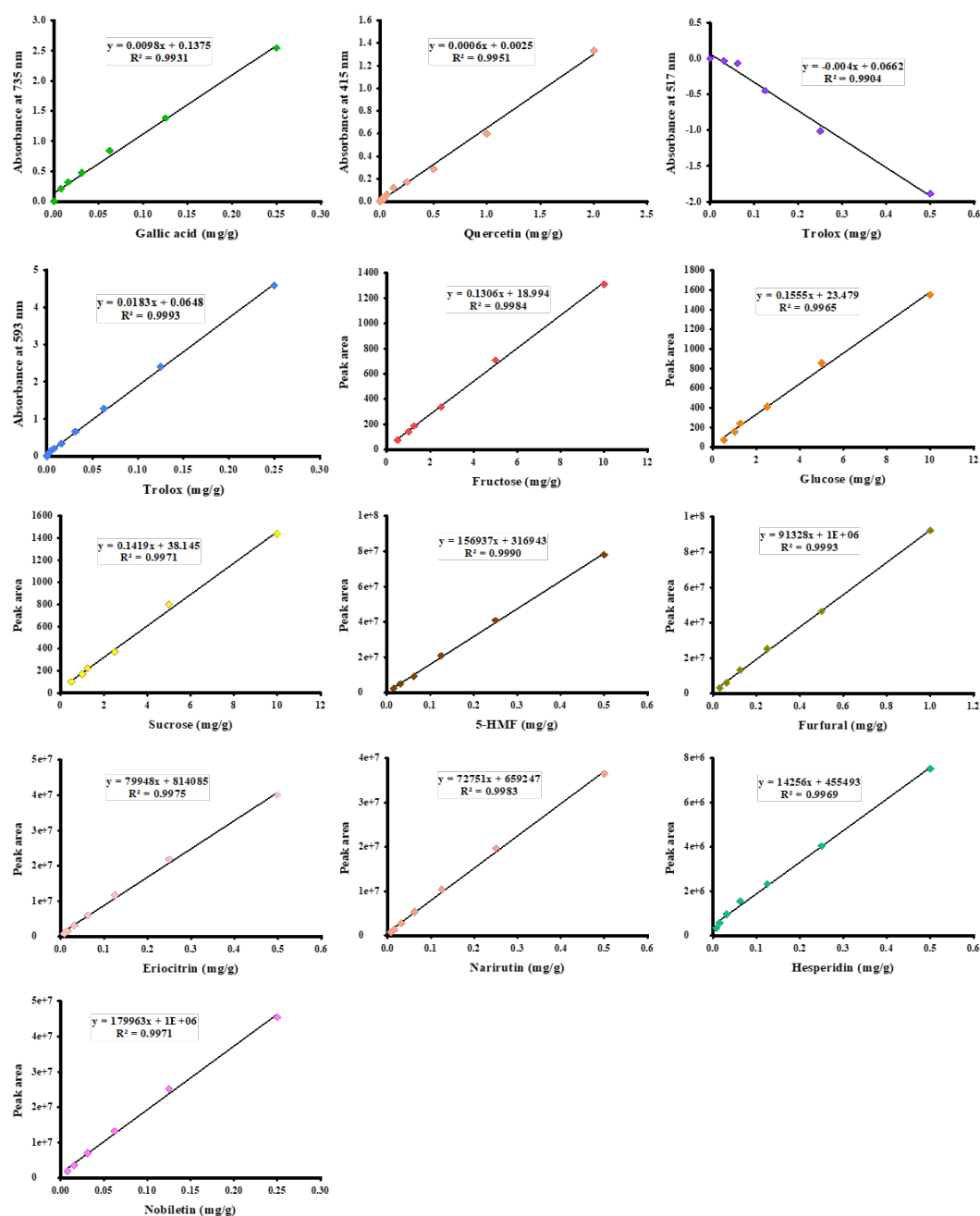

Figure S1. Calibration curve of standards.

Supplement: Supplementary file 1 [file foods-13-03053-s001.zip › foods-3223857-supplementary.pdf]
